# Brome mosaic virus-like particles as siRNA nanocarriers for biomedical purposes

**DOI:** 10.3762/bjnano.11.28

**Published:** 2020-02-20

**Authors:** Alfredo Nuñez-Rivera, Pierrick G J Fournier, Danna L Arellano, Ana G Rodriguez-Hernandez, Rafael Vazquez-Duhalt, Ruben D Cadena-Nava

**Affiliations:** 1 Centro de Nanociencias y Nanotecnología - Universidad Nacional Autónoma de México (UNAM) – Ensenada, Baja California, México; 2 Centro de Investigación Científica y de Educación Superior de Ensenada, Baja California, (CICESE), Ensenada, Baja California, México

**Keywords:** anti-cancer therapy, brome mosaic virus (BMV), cowpea chlorotic mottle virus (CCMV), nanocarriers, plant virus-like particles (VLPs), siRNA delivery, small interfering RNA (siRNA)

## Abstract

There is an increasing interest in the use of plant viruses as vehicles for anti-cancer therapy. In particular, the plant virus brome mosaic virus (BMV) and cowpea chlorotic mottle virus (CCMV) are novel potential nanocarriers for different therapies in nanomedicine. In this work, BMV and CCMV were loaded with a fluorophore and assayed on breast tumor cells. The viruses BMV and CCMV were internalized into breast tumor cells. Both viruses, BMV and CCMV, did not show cytotoxic effects on tumor cells in vitro. However, only BMV did not activate macrophages in vitro. This suggests that BMV is less immunogenic and may be a potential carrier for therapy delivery in tumor cells. Furthermore, BMV virus-like particles (VLPs) were efficiently loaded with small interfering RNA (siRNA) without packaging signal. The gene silencing was demonstrated by VLPs loaded with siGFP and tested on breast tumor cells that constitutively express the green fluorescent protein (GPF). After VLP-siGFP treatment, GFP expression was efficiently inhibited corroborating the cargo release inside tumor cells and the gene silencing. In addition, BMV VLP carring siAkt1 inhibited the tumor growth in mice. These results show the attractive potential of plant virus VLPs to deliver molecular therapy to tumor cells with low immunogenic response.

## Introduction

Despite many efforts taken, the efficient and specific delivery of therapeutic molecules to tumor cells is still a unsolved challenge. Cancer therapies are often limited because only a small fraction of the administered dose of the drug arrives into the tumors [1–3]. This can be attributed, in part, to a series of biological barriers that reduce the drug accumulation in tumors [4] such as sequestration by the mononuclear phagocyte system [5], non-specific distribution [6], limitations in the blood flow of tumor vessels [7], pressure gradients, cellular internalization [8], and the escape of endosomal and lysosomal compartments and drug efflux pumps [9].

The use of nanoparticles as nanovehicles has been proposed to overcome some of these limitations. Nanoparticles offer several advantages such as their size and a surface area that could be functionalized with specific ligands in order to be targeted to specific tissues [10]. Additionally, they can be used to increase the overall solubility of drugs and to modulate their circulation half-life [11–13]. The accumulation of nanoparticles in tumors, either passively or directed, is extensively documented [14]. Thus, there are multiple efforts to design nanoparticles that function as nanovehicles, mainly composed of liposomes, synthetic polymers, dendrimers, and virus-like particles (VLPs) [13,15]. Recently, the use of VLPs with high loading capacity and biocompatibility has reached clinical stages [16–17].

Plant virus VLPs have received less attention, since for most of the viral vector developments bacteriophages and complex mammalian viruses are used. However, due to their easy production, handling, and simple structure, plant viruses are attractive for some biomedical applications. Plant bromoviruses, such as the brome mosaic virus (BMV), are viral bionanoparticles that have been proposed as platforms for drug delivery in different therapies, and as diagnostic imaging agents in cancer [18–20]. The capsids of these viruses result from the assembly of 180 identical proteins with *T* = 3 symmetry that forms the icosahedral shell with a diameter of 28 nm [21]. The N-terminal region of the capsid protein is highly basic and positively charged, which allows for the binding of the viral RNA genome [22]. Also, the casid protein able to encapsidate anionic molecules, such as heterologous RNAs [23], enzymes [24], drugs [25], or gold nanoparticles [26] by charge complementarity with the possibility of directing them to target cells through the functionalization of the external surface of the capsid [25,27]. Similarly, VLPs from the closely related cowpea chlorotic mottle virus (CCMV) have been loaded with different cargos, including gold nanoparticles, negatively charged chromophores, and polymers [22].

Small interfering RNA (siRNA) is a promising therapeutic solution to address gene overexpression or mutations for several pathological conditions such as viral infections, cancer, genetic disorders, and autoimmune disorders such as arthritis [28–29]. Especially, BMV VLPs show potential for delivering siRNA due to easy production and purification, and high stability. Also, they can be readily modified chemically and genetically for potential therapeutic applications [30].

Despite these interesting properties, to our knowledge, the use of BMV VLPs for molecular cancer therapies, especially in delivering siRNA for gene silencing, has been scarcely explored. In this work, important BMV VLPs properties of biomedical interest are demonstrated, such as biocompatibility, tumor cell internalization, and their efficiency as nanocarriers for siRNA delivery. In addition, the capacity of the BMV and CCMV viruses to modulate the immune response in vitro was also analyzed.

## Results and Discussion

### Cell internalization of VLPs

**Figure 1:**
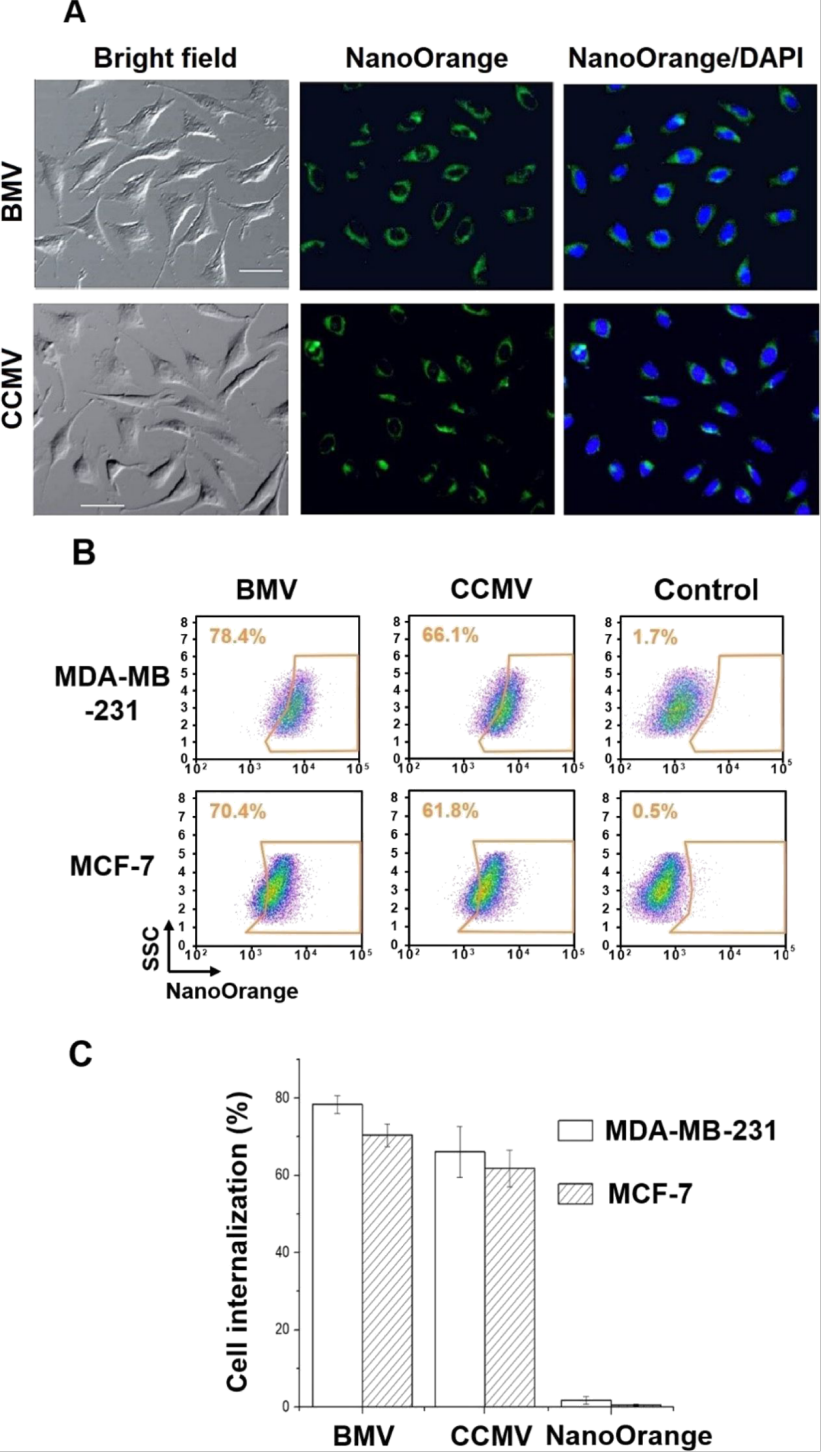
Cellular uptake of CCMV and BMV viruses. (a) Confocal laser scanning microscopy (CLSM) images of MCF-7 cells treated with virus–NanoOrange (green channel). (b) Representative flow cytometry data (c) and the statistical data of virus internalization in MDA-MB-231 and MCF-7 tumor cells. Free NanoOrange (controls) was virtually not internalized. Scale bar = 50 μm. Error bars represent mean ± SD (*n* = 3).

In order to test the cell internalization of VLPs, BMV and CCMV VLPs were loaded with NanoOrange, a hydrophobic fluorescent dye. Both BMV and CCMV viruses have hydrophobic residues in their capsid protein in which hydrophobic molecules, such as NanoOrange, are bound. Due to the high fluorescence of this fluorophore, the internalization into tumor cells using this labeling technique appears to be better when compared with some previous reports [31–33]. The NanoOrange-loaded BMV and CCMV capsids were then incubated in MCF-7 cell cultures for 4 h to evaluate their internalization into the breast cancer cells. Representative confocal microscopy images showed NanoOrange fluorescence inside the cells (Figure 1A). It is important to point out that the capsids are able to internalize efficiently into tumor cells without any functionalization. The cell internalization has been quantified by flow cytometry (Figure 1B). The differences in the extent of cell internalization could be explained by the surface charge as revealed by zeta potential measurements. The flow cytometry analysis of cell internalization was also performed after trypsin treatment, which promotes detachment of the capsids from the cell surface [31], and thus only internalized capsids are detected.

**Figure 2:**
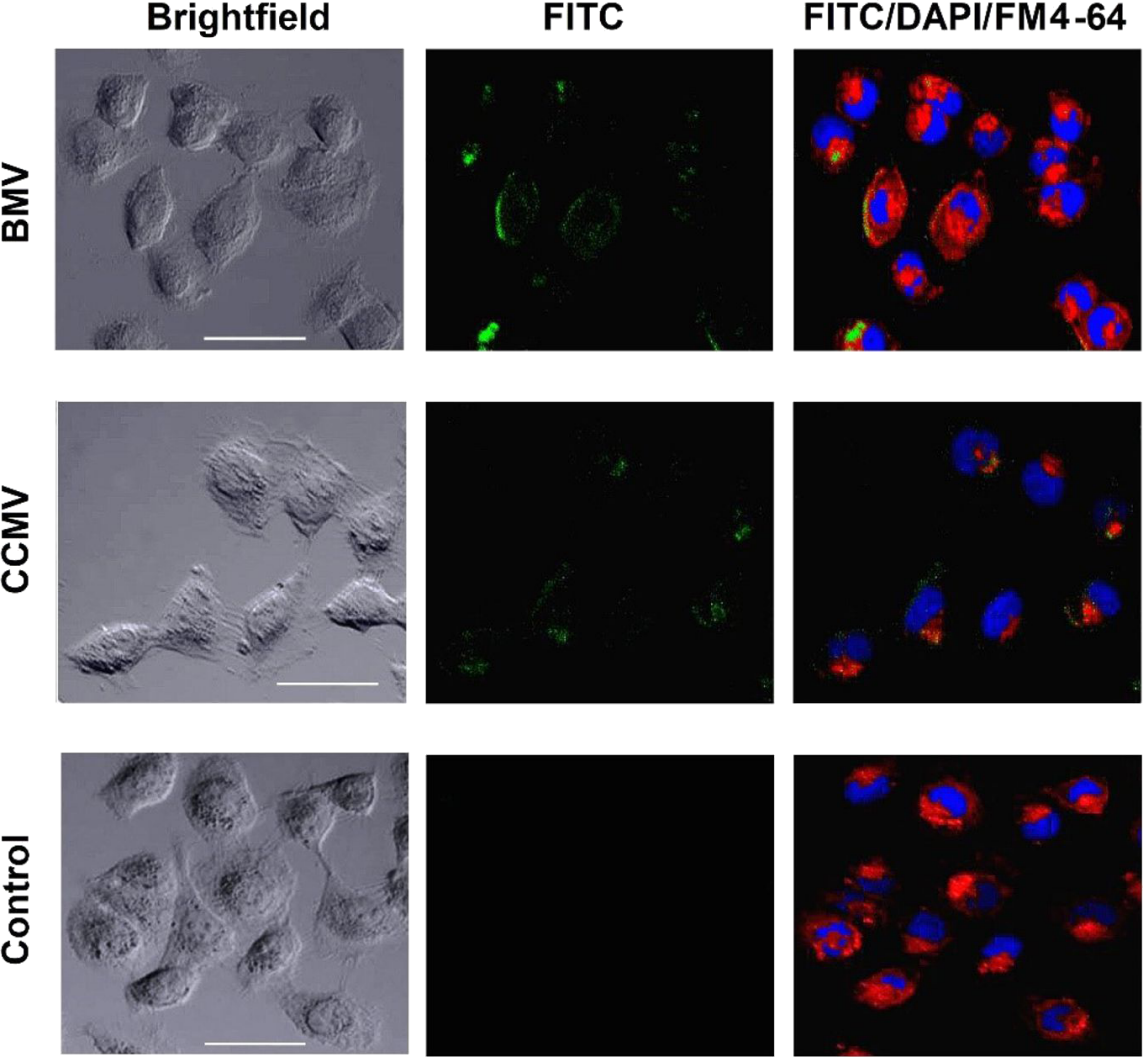
Internalization of CCMV and BMV conjugated with FITC. MDA-MB-231 cells were incubated with BMV-FITC (green channel) and CCMV-FITC (green channel). The cell nucleus was stained with DAPI (blue channel) and the membrane with FM4-64 (red channel). Free FITC did not show internalization. A concentration of 1.3 × 10^6^ viruses/cell was used. Scale bar = 50 μm.

The results showed a slightly higher efficiency of internalization, but no significant difference, for BMV into the MDA-MB-231 and MCF-7 breast tumor cell lines (Figure 1C). Furthermore, to avoid erroneous results due to possible detachment of NanoOrange from the capsids, FITC fluorophore was covalently conjugated to the capsid surface and analyzed by confocal microscopy (Figure 2 and Figure S1, Supporting Information File 1). The confocal images showed FITC fluorescence inside the cells without colocalization of the plasma membrane stained with FM4-64, evidencing an effective cell internalization of both BMV and CCMV loaded capsids. With the virus loaded with FTIC a similar efficiency of virus internalization in MDA-MB-231 and MCF-7capsids are interacting with the cell plasma membrane, in cells was observed. Flow cytometry analysis showed a 70% virus internalization in the cells treated with both CCMV and BMV capsids (Figure 1B,C). In addition, confocal microscopy images showed that almost all the cells contained plant viruses with loaded NanoOrange or with covalently conjugated FITC (Figure 1A and Figure 2). The differences in fluorescence intensity detected by confocal microscopy among the treated cells can be attributed to the different amounts of internalized VLPs.

We demonstrate that the BMV and CCMV capsids are efficiently internalized by breast tumor cells, without the need to couple a specific cell ligand. These results differ from those reported for cell internalization of cowpea mosaic virus (CPMV) in the same cell lines, which do not show high levels of vimentin on the cell surface [20,34]. Vimentin is a receptor that facilitates caveolar endocytosis [35–36], and it has been reported to promote the cellular internalization of CPMV [32,35–37]. Therefore, the cellular internalization of the CPMV virus occurs via endocytosis, which includes multiple routes: clathrin-dependent, caveolar and micropinocytosis [36–38]. Our results showed that the presence of low levels of vimentin on the cell surface is not a limiting factor for the cell internalization of BMV and CCMV. Thus, it seems possible that the capsid internalization could be carried out by macropinocytosis, a process independent of vimentin.

### Biocompatibility of CCMV and BMV

**Figure 3:**
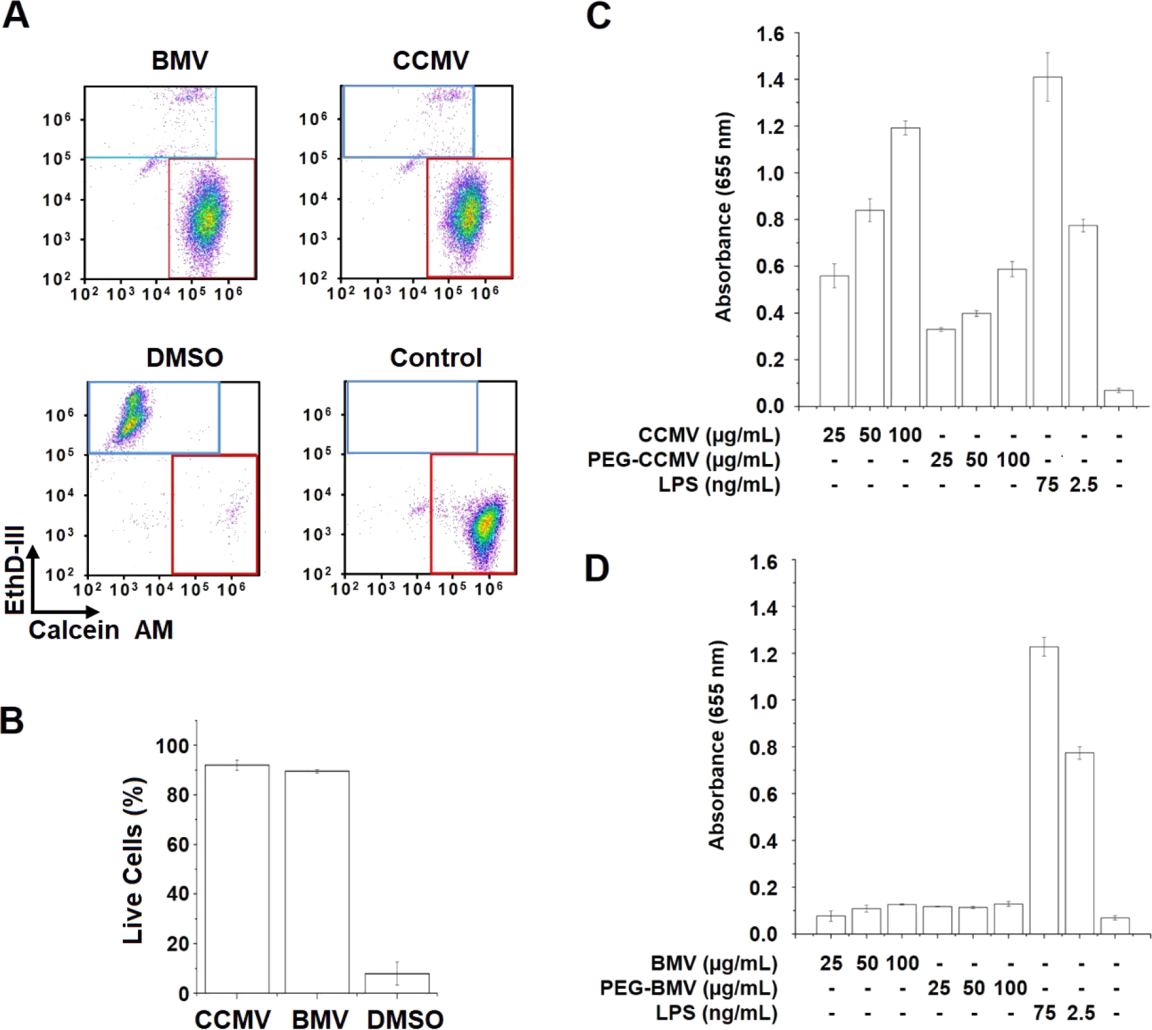
Biocompatibility and immune response of BMV and CCMV. (A) MDA-MB-231 cells were incubated with the virus CCMV and BMV for 24 h with 2.6 × 10^7^ viruses/cell. The cytotoxicity was measured by flow cytometry, using calcein AM to quantify live cells and EthD-III for dead cells. (B) Representative statistical data of live cells after virus incubation. Macrophage activation by (C) CCMV, (D) BMV and PEGylated virus nanoparticles (PEG-CCMV/PEG-BMV) at different concentrations were measured using RAW 264.7-Blue cells. Lipopolysaccharide extract (LPS) was used as a control of macrophages activation. Error bars represent mean ± SD (*n* = 3).

A possible virus cytotoxicity was evaluated. BMV and CCMV viruses were incubated for 24 h with MDA-MB-231 cells, using 2.6 × 10^7^ viruses per cell. A similar concentration has been used in cell viability tests with CCMV [25,39] and glycol chitosan nanoparticles [40]. The flow cytometry results showed around 90% cell survival after treatment with both viruses (Figure 3A,B), while almost all cells died after treatment with DMSO (death control). These results agree with previous studies with BMV VLPs in HBE cells [41] and also with other plant viruses, in which even at high virus concentrations, no cytotoxic effect on cells was found [38,42–43]. This high degree of biocompatibility make plant viruses capsids suitable candidates as carriers to deliver therapeutic drugs or siRNA molecules.

### Immunogenicity of CCMV and BMV

The RAW 264.7-blue macrophage cell line was used to determine the potential immune response in vitro of both BMV and CCMV viruses. Surprisingly, a remarkable difference was found. CCMV showed a high activation of macrophages, while BMV showed almost no immunogenic response (Figure 3C,D). There is 80% homology in the amino acids sequences of CCMV and BMV [21], however, they differ in their surface charge. The zeta potential at pH 7 was determined. Under these conditions, the zeta potential of CCMV is −9.27 ± 0.47 mV, more negative than that of BMV (−5.16 ± 0.40 mV). The surface charge of the capsid could be the reason why CCMV activates macrophage cells to a greater extent, because it is well known that at a higher anionic charge the particles tend to be phagocytosed by macrophages [44]. Accordingly, the virus uptake by the macrophages can activate intracellular receptors, i.e., toll-like receptors (TLR) 7/8, which can recognize the viral ssRNA genome, promoting the activation of the macrophages. This mechanism of TLR 7/8 activation has been reported using papaya mosaic virus [45].

Both capsids of CCMV and BMV were covered with polyethylene glycol (PEG) to decrease their surface charge and mask the domains of the capsid proteins that could be recognized by the macrophages (Figure 3C,D). PEG is widely used to reduce the immunogenicity of proteins. Also, PEG has been approved by the US Food and Drug Administration, and there are now several PEGylated drugs commercially available. The PEG–drug conjugates show several advantages that include prolonged residence in the body, reduced degradation by metabolic enzymes, and reduced or no protein immunogenicity [46]. Although the PEGylation of CCMV capsids (CCMV-PEG) greatly reduced the immunogenic response, BMV seems to be a better nanocarrier candidate due to its low immunological response. On the other hand, and despite of the immunogenicity of CCMV, which can limit its use for certain therapies, this virus could act as an immunoregulator in immunological therapies to improve some cancer treatments [47–48].

### VLP-siRNA synthesis and characterization

Gene silencing through RNA interference (RNAi) is considered to be among the most promising therapies to fight cancer. RNAi pathway influences the translation of mRNA through silencing specific genes in nearly all human cells [49]. The use of siRNA in RNAi has been limited so far by the lack of a vehicle that delivers siRNA to the desired tissue. To solve this problem, we are proposing to use plant viral capsids as nanocarriers to deliver siRNA. VLPs nanovehicles containing siRNA were synthesized, taking advantage of the self-assembly of BMV capsid proteins.

**Figure 4:**
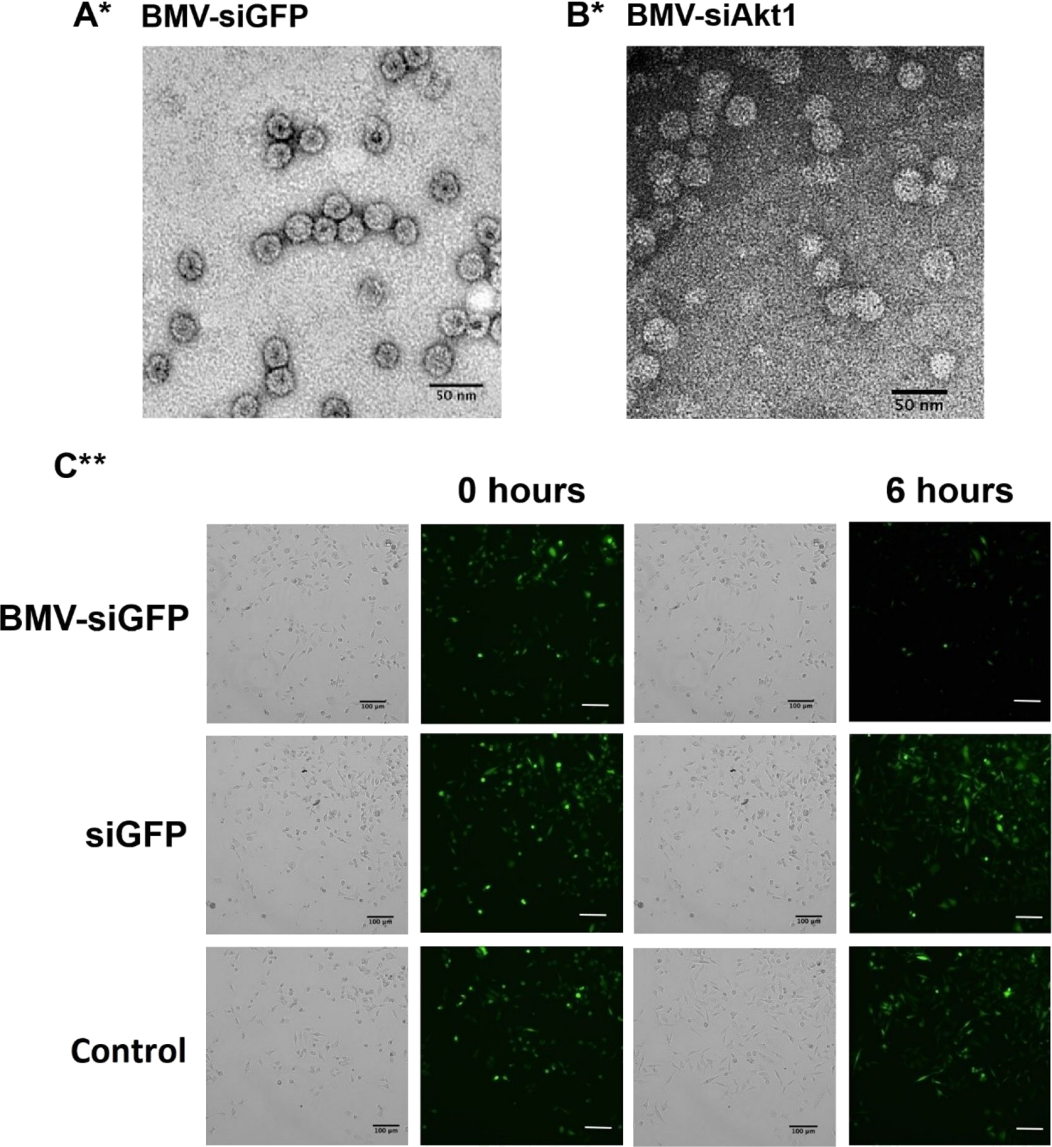
Encapsidation of siRNA in BMV capsid proteins. TEM images of virus-like particles in vitro assembled at a mass ratio of 1:6 (siRNA/CP BMV). (A) BMV VLP-siGFP, (B) BMV VLP-siAkt1. (C) GFP silencing assay using human breast tumor cells MDA-MB-231 that stably express GFP. The cells were incubated with BMV VLP-siGFP and siGFP at a concentration of 50 nM. *Scale bar = 50 μm, **scale bar = 100 μm.

As a proof of concept, siRNA to silence the expression of green fluorescent protein (GFP) was encapsidated into BMV capsid proteins. In order to optimize the siRNA encapsidation, different ratios of siGFP and CP were examined, monitoring the uncapsidated GFP. An efficient encapasidation of siRNA to form the VLP-siGFP nanocarriers was obtained using a mass ratio of 1:6 (siGFP/CP). The VLP-siRNA from BMV showed icosahedral VLPs of approximately 27.7 nm in diameter, similar to those of the native virus (*T* = 3), as corroborated by TEM (Figure 4A and Figure S2a, Supporting Information File 1). Importantly, this is the first report of the encapsidation of nucleic acids in VLPs from BMV without the need for an RNA packaging signal. Previous reports showed that a tRNA-like structure is crucial to assemble BMV-VLPs with nonviral RNA [50–51].

The BMV VLPs containing siGFP were incubated in breast tumor cells MDA-MD-231, which constitutively express GPF. After VLP-siGFP treatment GFP expression was efficiently inhibited, as shown by the reduction in fluorescence after 6 h of treatment (Figure 4C), corroborating the cargo release from BMV VLP inside tumor cells and gene silencing.

### BMV VLPs as siAkt1 nanocarriers

The anti-cancer siRNA Akt1 (siAkt1) was also encapsidated in BVM-VLPs (Figure 4B). Akt1 is a kinase involved in the processes of cell proliferation, migration and transformation [52–55]. The siAkt1 encapsidation was performed using a mass ratio of 1:6 (siRNA-Akt1/CP-BMV). VLPs with icosahedral morphology of around 27.8 nm of diameter (*T* = 3) were obtained, similar to the VLP-siGFP (Figure 4 and Figure S2b, Supporting Information File 1) and the wild-type BMV (29.3 nm). The nanoparticle diameters were in agreement with those from DLS measurements, see Figure S3 (Supporting Information File 1). The amount of BMV capsid protein and siRNA was quantified by fluorescence assays. The correlation between the observed fluorescence and the concentration allows for the estimation of the average concentration of capsid protein and siRNA in the BMV-VLPs suspension (see Figure S4, Supporting Information File 1). With this estimation, together with the data from TEM measurements, an average of 100 µg of CP for 23.8 µg of siRNA was estimated, i.e., an average of 66 siRNA molecules in the *T* = 3 capsid (180 CP molecules). Thus, the siRNA confinement in BMV-VLPs is 10 times higher than that reported for CCMV VLPs [32]. Considering that siRNA has 42 e^−^ charges, there are 2,772 negative charges inside of the VLP, which is in agreement with the average of negative charges in the wild-type BMV: 3000 e^−^ /capsid [21–22].

**Figure 5:**
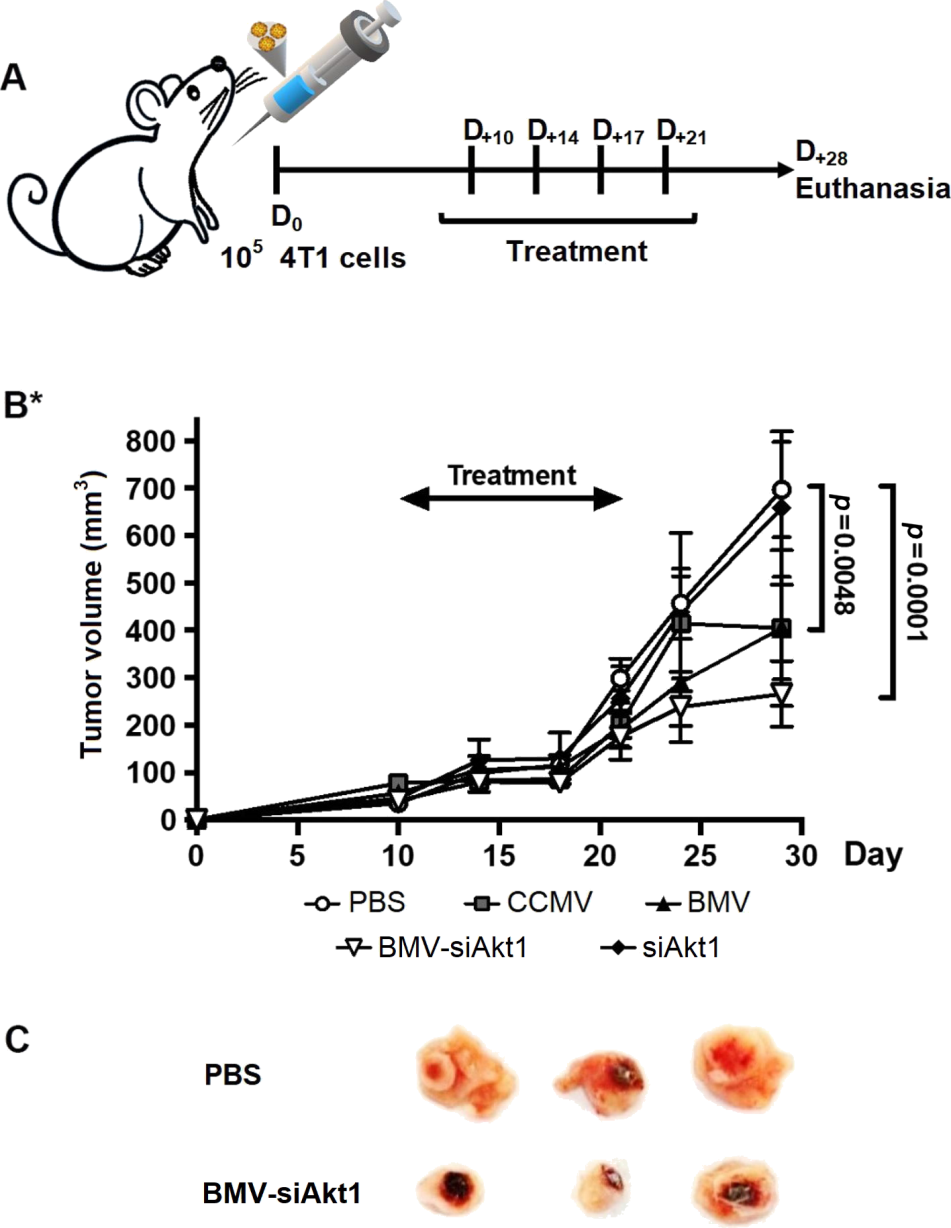
Anti-tumor effect of virus nanoparticles. (A) Schematic illustration of the experimental design. (B) In vivo growth curves of 4T1 tumors on mice after treatments with the virus and VLPs. (C) Ex vivo images of 4T1 breast cancer tumor from BMV VLP-siAkt1 and PBS control treatments on day 28 after tumor challenge. BMV VLP-siAkt1 showed the strongest inhibition effect on the tumor compared to the other nanoparticles. Error bars represent mean ± SD. *P*-values were calculated by 2-way ANOVA with Tukey posttest (*P < 0.01).

The synthesized VLP-siAkt with both BMV and CCMV capsids were assayed in vivo. The VLP-siAkt were inoculated into mice with previously induced breast cancer tumors. The tumors were induced in female mice using the mouse cell line 4T1. After ten days of tumor inoculation, doses of 100 µg of the plant virus and VLP-siRNA were administered by injection every three days for two weeks in the periphery of the tumor (Figure 5A). The size of the tumor was evaluated twice a week for 28 days. Significant differences among the treatments were detected (Figure 5B). BMV-siAkt1 showed the highest activity, inhibiting around 50% of tumor size when compared with the control (Figure 5B,C). The efficiency of BMV-VLPs in delivering siRNA is similar to that observed when nanoparticles of multilamellar gold niosomes were used to deliver siRNA-Akt [56]. The mice showed no significant weight differences after the different treatments, suggesting that the VLPs and viral nanoparticles do not alter the mice metabolism, which could influence tumor growth. The slight tumor growth reduction induced by the treatment with the wild type virus could be attributed to their immunogenic capacity. These results are in agreement with the in vitro macrophage activation experiments showed above for CCMV, and also with previous reports on the success of in situ immunoregulation of tumors and the inhibition of metastasis using VLPs of the CPMV virus [57]. It is well known that treatments with CPMV-VLPs show greater inhibition of the tumor than using a highly immunogenic agent such as lipopolysaccharide (LPS), poly (I: C), and DMXAA 47. It seems that CCMV regulates the tumour microenvironment similarly to the CPMV virus [57]. The virus could induce the polarization of macrophages by recognizing intracellular toll-like receptors [45,58–59], or the expression of cytokines and chemokines, which activate infiltrated neutrophils in the tumor producing reactive oxygen species (ROS) [60]. The virus could also modulate and recruit CD8 + T cells, and natural killer cells to generate a cytotoxic effect [60–61]. However, the results obtained with the wild-type BMV are still unclear. Finally, our in vivo results demonstrated that BMV-VLPs nanocarriers containing siRNA are able to be internalized by the tumor cells and deliver the siAkt1 cargo into the tumor.

## Conclusion

The capacity of BMV VLPs to carry and deliver siRNA into tumor cells has been demonstrated. Cell internalization of the plant viruses, BMV and CCMV, showed no cytotoxicity, making the viruses excellent and biocompatible nanocarrier candidates for targeted molecular anti-cancer therapies. BMV-based nanocarriers showed better efficiency in cell transfection without inducing in vitro immunological responses when compared with CCMV. In addition, the efficient synthesis of icosahedral BMV VLP-siRNA and its ability to release the small interference RNA molecules into tumor cells was also demonstrated. Thus, the BMV capsids are a potential candidate to deliver siRNA. The versatility of these plant virus nanocarriers includes the functionalization of the viral capsid surface with specific ligands, e.g., folic acid, antibodies, or modified mannose, which further increase the recognition of specific targeted tissues or tumor cells. The coupling of drugs and molecular therapies, such as siRNA, in the same nanocarrier seems to be an excellent strategy to increase the efficiency of anti-cancer therapies.

## Experimental

### Production and purification of the virus

CCMV and BMV were obtained from infected cowpea and barley plants, respectively. The viruses were purified as described previously [22]. Briefly, one week after germination the plant leaves were slightly damaged mechanically to be able to be infected with the wild-type virus, using the inoculation buffer (0.01 M sodium phosphate buffer, pH 6 and 0.01 M magnesium chloride) containing 0.1 μg/μL of wild-type virus suspension. After the plants showed symptoms of infection the leaves were collected, chopped in a blender with extraction buffer (0.5 M sodium acetate and 0.08 M magnesium acetate, pH 4.5). The mixture was filtered through cheesecloth and then one volume of chloroform was added and the mixture was kept under stirring at 4 °C. To recover the aqueous phase, the mixture was centrifuged at 12,300*g* for 15 min at 4 °C. The aqueous phase was kept under stirring for 2 h and placed on a 10% sucrose cushion to separate the protein fraction. The cushion was ultracentrifuged at 110,000*g* for 120 min using a Beckman SW 32 Ti rotor in a Optima XPN-100 ultracentrifuge. The formed pellet was resuspended with virus suspension buffer and the virus was ultrapurified using a sucrose gradient, which was ultracentrifuged at 110,000*g* at 4 °C for 120 min. The concentration of the virus was calculated by measuring the UV absorbance using a Nanodrop 2000c spectrophotometer (ThermoFisher Scientific). All the procedures were performed at 4 °C. Finally, the purified viruses were stored at −80 °C in 1.5 mL microcentrifuge tubes.

#### Cell cultures

The breast tumor cell lines MDA-MB-231 and MCF-7 were obtained from the ATCC. The cell line MDA-MB-231/GFPm which constitutively expresses green fluorescent protein (GFP) was obtained from Cell Biolabs. RAW-Blue (InvivoGen) was derived from the mouse macrophage cell line RAW 264.7 after adding the sequence of the secreted embryonic alkaline phosphatase (SEAP) under the control of an NF-κB/AP-1 inducible promoter. RAW-Blue cells can be used as pattern-recognition receptor reporter cells to assess macrophage activation. All these cells were cultured with high-glucose DMEM media (Biowest). The breast-tumor cell line 4T1 (ATCC) was derived from a spontaneously arising mammary tumor from a MMTV+ Balb/C mice and forms tumors when implanted in the mammary fat of Balb/C mice. The 4T1 cells were cultured in RPMI medium (Corning). DMEM and RPMI basal media were supplemented with 10% fetal bovine serum (FBS, Biowest) and antibiotic/antimycotic (ThermoFisher Scientific). The medium for MCF-7 cells was also supplemented with recombinant human insulin (0.01 mg/mL; Sigma-Aldrich) and the medium of the RAW-Blue cells with 100 µg/mL of Normocin and 200 µg/mL of Zeocin antibiotics (InvivoGen). Cells were maintained at 37 °C in a humidified incubator with 5% CO_2_.

#### Virus cell internalization

To visualize the cell internalization, viruses were loaded with NanoOrange. These are hydrophobic molecules that bind to the hydrophobic domains of the capsid proteins of BMV and CCMV. NanoOrange (NanoOrange™ Protein Quantitation Kit; ThermoFisher Scientific) were incubated with 1 µg of BMV or CCMV capsids for 20 min. The virus capsids were also rendered fluorescent by the covalent conjugation of the free amino groups of proteins with fluorescein-5-isothiocyanate (FITC). The FITC conjugation was carried out according to Douglas et al. [27] as follows: 400 µg of viral capsid were labeled in a solution of FITC (10 µg/mL, Molecular Probes) in PBS, under constant agitation, for 2 h at room temperature. The excess of the fluorophore was removed by ultrafiltration using 100 kDa amicon filters (0.5 mL, Millipore). For the controls, free NanoOrange and FITC were utilized at the same concentration to label the viruses.

A suspension containing 10^5^ MDA-MB-231 or MCF-7 cells was seeded in 35 mm glass bottom dishes (MatTek Co.) or in 12-well plates. After 12 h, the medium was completely removed and cells were incubated for another 12 h in basal media. BMV or CCMV viral capsids labeled with NanoOrange, or FITC were added to the culture (1.3 × 10^6^ virions per cell) and incubated for 4 h. The amount of virus used is consistent with previous studies with plant viruses [32,35–37,41]. Virus internalization was monitored on live cells or cells fixed with paraformaldehyde, after staining the membrane with FM4-64 (ThermoFisher Scientific) and the nucleus with DAPI (Sigma-Aldrich), using a confocal microscope FV1000 FluoView (Olympus). Additionally, cells were trypsinized, centrifuged (800*g*, 5 min) and resuspended in PBS before analyzing virus internalization using an Attune acoustic focusing cytometer (Thermo Fisher Scientific). Doublets were excluded by plotting the height as a function of the area for the forward scatter and then of the side scatter. Debris were then excluded by plotting the area of the forward scatter as a function of the area of the side scatter. For each sample, a least 7,500 events corresponding to single cells were collected and the Attune Cytometric Software (v2.1) was used to analyze results. For the controls, the same amount of free NanoOrange was used as the one used to load viruses.

#### Cell viability assay

MDA-MB-231 cells were seeded in 12-well plates, as described previously. When the cell monolayers reached about 70% confluence, the medium was completely removed and cells were synchronized in basal media for 12 h. The cells were then further incubated in the presence of BMV or CCMV (20 µg/mL, 2.6 × 10^7^ virions per cell) or DMSO (20% v/v) as a control for cell death and PBS as a control medium for 24 h. The Viability/Cytotoxicity Assay Kit (Biotum) was used to assess the amount of live and dead cells. Briefly, cells were trypsinized and labeled with calcein and ethidium homodimer III (EthD-III) to recognize live and dead cells, respectively. Cells were analyzed by flow cytometry. For each sample at least 7,500 events corresponding to single cells were collected as described previously, and the Attune Cytometric Software (v2.1) was used to analyze results.

#### Surface functionalization of BMV and CCMV with PEG

The external surface of the capsid was PEGylated using polyethylene glycol functionalized with *N*-hydroxylsuccinimide (NHS-PEG, Sigma Aldrich). The use of NHS-PEG enabled the selective conjugation of nanoparticles with PEG via amide bonds. A solution of NHS-PEG (9.12 nmol) was mixed with the capsids suspension (1 mg/mL) in PBS and gently shaken for 2 h at room temperature. The excess of PEG was removed by ultrafiltration using Amicon filters (Millipore) of 100 kDa cutoff.

#### In vitro immunogenicity of CCMV and BMV viruses

The macrophage cell line RAW-Blue was used to evaluate the in vitro immunogenicity. Experiments were performed according to the manufacturer’s guidelines. Cells at 80% confluence were washed twice with sterile PBS, manually detached by scraping and resuspended in fresh medium. Live cells were seeded in 96-well plates (105 per well) and incubated overnight in the presence or absence of CCMV or BMV (20 to 100 µg/mL, 2.6 × 10^7^ to 1.3 × 10^8^ virions per cell), PEGylated or non-functionalized capsids, or as positive control lipopolysaccharides (2.5 or 75 ng/mL). Then, 50 µL of supernatant was transferred into a new 96-well plate and 150 µL of Quantity Blue (InvivoGen) reagent was added and incubated at 37 °C for 1 h. The absorbance at 655 nm was measured using a Multiskan GO plate reader (ThermoFisher Scientific).

#### Synthesis and characterization of BMV VLP-siRNA

siRNA against GFP and mouse Akt1 were purchased from Dharmacon. The sequences of the siRNA against GFP (siGFP) was 5′-GGCAAGCUGACCCUGAAGUUCAUU-3′ and against Akt1 (siAkt1) was 5′-GACAAGGACGGGCACAUUAUU-3′. BMV VLPs with siRNA were synthetized as described previously [22] using a mass ratio of 1:6 (siRNA/capsid protein). The disassembled BMV capsid protein and siRNA were mixed and dialyzed overnight in an assembly buffer (50 mM NaCl, 10 mM KCl, 5 mM MgCl_2_, 1 mM DTT, 50 mM Tris-HCl, pH 7.2), followed by dialysis against acidification buffer (50 mM sodium acetate, 8 mM magnesium acetate, pH 4.5) for 8 h and finally the sample was dialyzed in assembly buffer. All dialysis experiments were performed at a temperature of 4 °C using a dialysis membrane with a 14 kDa cut-off (Spectrumlabs).

The morphology of the VLP-siRNA was evaluated by transmission electron microscopy. Copper grids (400 mesh, Ted Pella) were used, in which 6 μL of the sample was placed at a concentration of 0.1 μg/μL and after two minutes the liquid excess was removed by using Whatman No. 2 filter paper. Negative contrast was obtained by the addition of 6 μL of 1% uranyl acetate for 1 min. The size and zeta potential of the viral nanoparticles and VLPs were determined by dynamic light scattering (DLS) using a Malvern NanoSizer.

The siRNA and the capsid protein were quantified from purified BMV VLP-siAkt1 using the Quant-it RiboGreen RNA assay (ThermoFisher Scientific) and NanoOrange protein quantification kit (ThermoFisher Scientific), respectively.

#### Knockdown of gene expression by VLP-siGFP

MDA-MB-231/GFP cells were seeded in a 12-well plate when the cells reached 70% confluence; they were synchronized in basal media overnight. Cells were then further cultured in complete medium containing 40 µL of BMV VLP-siGFP (50 nM of siGFP) for 6 h. The same concentration of free siGFP (50 nM) was used as a control. The amount of GFP was assessed using a LS720 fluorescence microscope (Etaluma).

#### Mouse model of mammary fat pad tumor

All animal experiments were performed in compliance with the local ethics committee of the Center for Scientific Research and Higher Education of Ensenada (CICESE). BALB/cAnNHsd female mice were obtained from Envigo. Mice were maintained in an Optimice cage system (Animal Care System), in a controlled environment room (temperature 24 °C and 12 h light/dark cycle) where they received water and food (2018 Teklad Global 18% protein rodent diet, Envigo) ad libitum. Mice were acclimated for at least a week before starting the experiments.

For the development of tumors, a cell suspension of 4T1 mouse breast cancer cells was prepared at a concentration of 2 × 10^6^ cells/mL in PBS. 4T1 cells were then inoculated in the left, upper (or 2nd) mammary fat pad (10^5^ cells in 50 µL) of 8-week old Balb/C female mice. One week after the inoculation, palpable tumors were detected in all the mice that were divided into four groups (*n* = 4) to receive CCMV, BMV or BMV capsids loaded with siAkt1 (100 µg of coat protein ) per mouse, 23.8 µg of free siAkt1 or PBS in the controls. They were inoculated intratumorally three times per week. Tumors were measured with a caliper three times and their size was calculated using the formula (*L*·*w*^2^)/2 where *L* and *w* stand for tumor length and width, respectively.

## Supporting Information

File 1 Internalization of CCMV and BMV in MCF-7 and characterization VLPs (DLS and TEM).
