# Supplementary material for: Brome mosaic virus-like particles as siRNA nanocarriers for biomedical purposes
Source: Beilstein J Nanotechnol. 2020 Feb 20;11:372–82. doi: 10.3762/bjnano.11.28 (PMC7059527; doi:10.3762/bjnano.11.28)
Supplement: File 1 — Internalization of CCMV and BMV in MCF-7 and characterization VLPs (DLS and TEM). [file Beilstein_J_Nanotechnol-11-372-s001.pdf]

BEILSTEIN JOURNAL OF NANOTECHNOLOGY

Supporting Information

for

Brome mosaic virus-like particles as siRNA nanocarriers for biomedical purposes

Alfredo Nuñez-Rivera, Pierrick G. J. Fournier, Danna L. Arellano, Ana G. Rodriguez-Hernandez, Rafael Vazquez-Duhalt and Ruben D. Cadena-Nava

Beilstein J. Nanotechnol. **2020,** *11,* 372–382. doi:10.3762/bjnano.11.28

Internalization of CCMV and BMV in MCF-7 and characterization VLPs (DLS and TEM)

License and Terms: This is a supporting information file under the terms of the Creative Commons Attribution License (https://creativecommons.org/licenses/by/4.0). Please note that the reuse, redistribution and reproduction in particular requires that the authors and source are credited.
The license is subject to the *Beilstein Journal of Nanotechnology* terms and conditions: (https://www.beilstein-journals.org/bjnano)

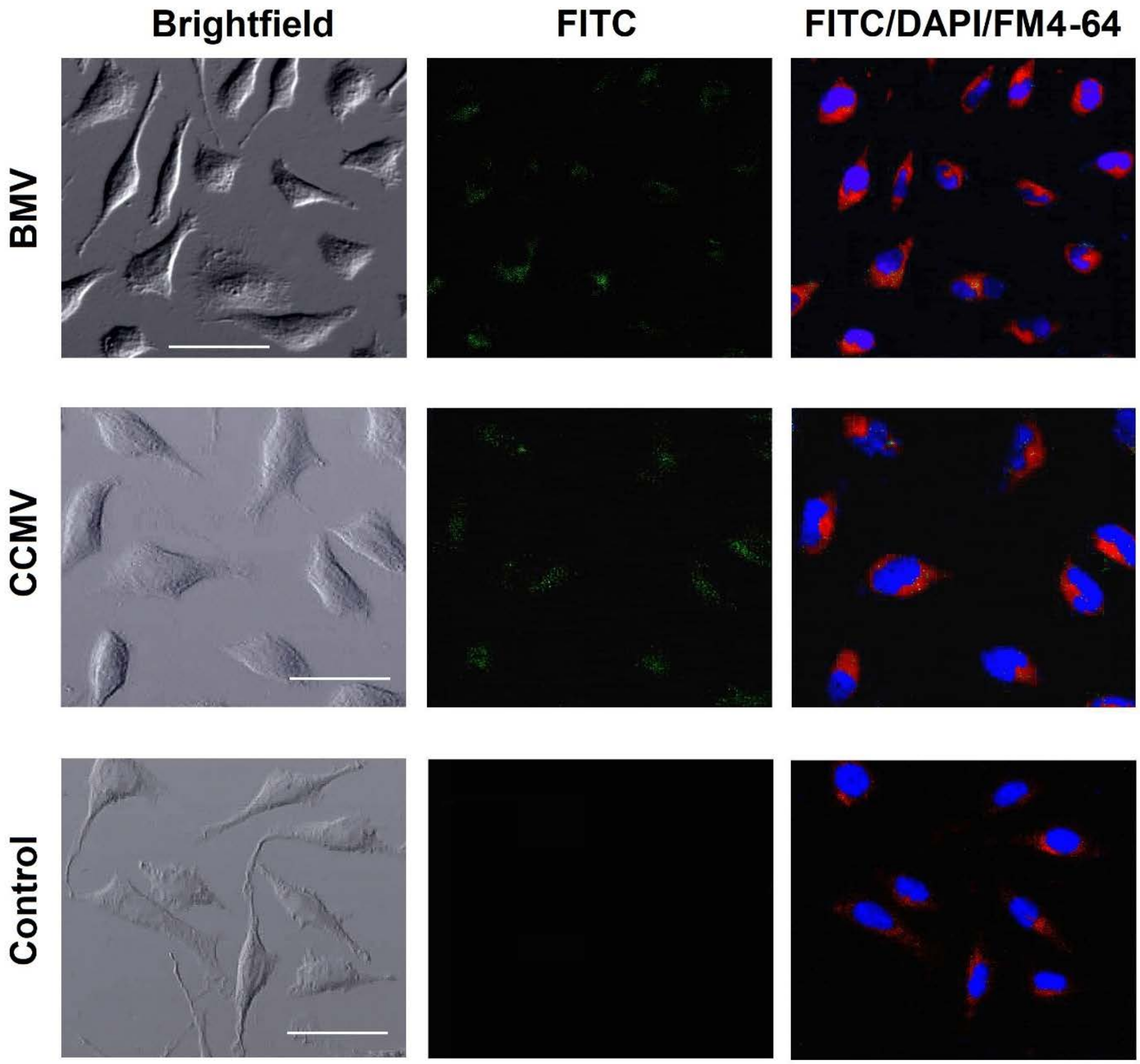

Figure S1: Internalization of CCMV and BMV conjugated with FITC. MCF-7 cells were incubated with BMV-FITC (green channel) and CCMV-FITC (green channel). The cell nucleus was stained with DAPI (blue channel) and FM4-64 for the membrane (red channel). A concentration of 1.3×10^6 virus/cell was used. Both viruses can be efficiently internalized by MCF-7 cells. Scale bar = 50 μm.

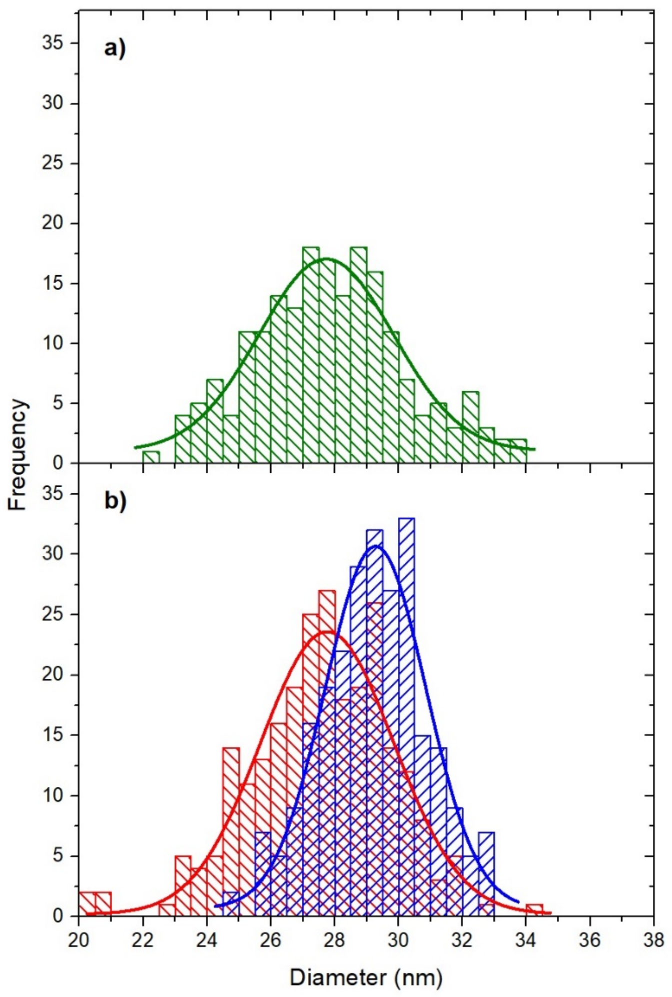

Figure S2: (a) Diameter histogram of BMV VLP-siGFP assembled using a mass ratio 6:1 CP to siGFP. The distribution has been determined from images of 200 capsids. (b) Diameter histogram of BMV VLP-siAkt1 assembled using the same mass ratio (CP to siRNA). The distribution has been determined from images of 250 capsids. Best-fit Gaussian curves, centered at 27.7 (green), 27.8 (red) and 29 nm (blue) for BMV VLPGFP, VLP-Akt1 and wild-type BMV respectively.

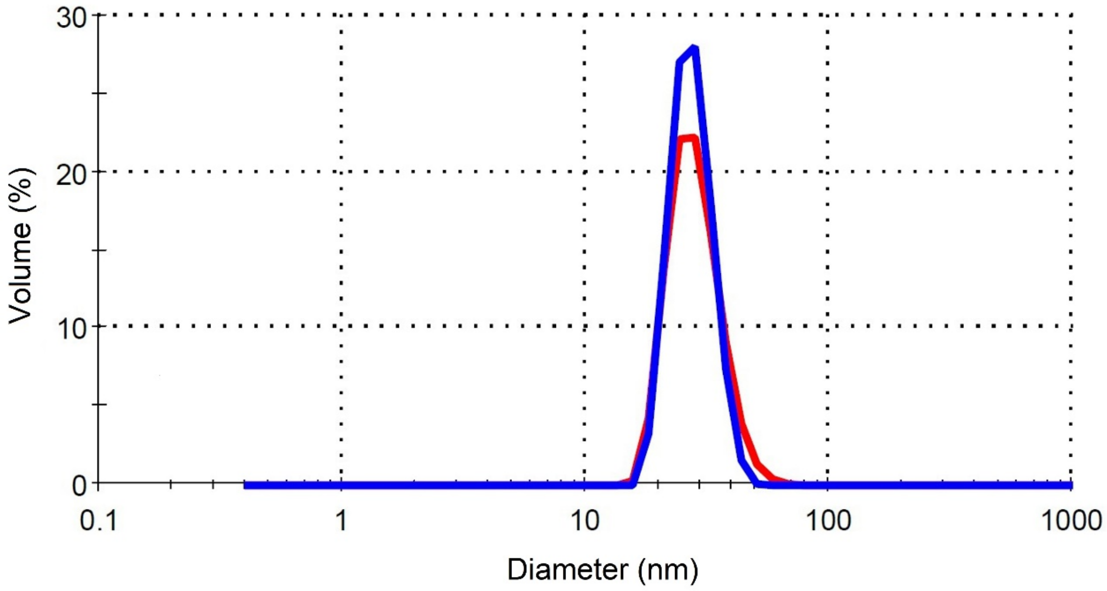

Figure S3: Hydrodynamic diameter distribution of BMV VLP-siAkt1 (red curve) and wild-type BMV (blue curve) by DLS. The size volume distribution histograms for VLP and wild-type BMV shows that the nanoparticles have about the same size.

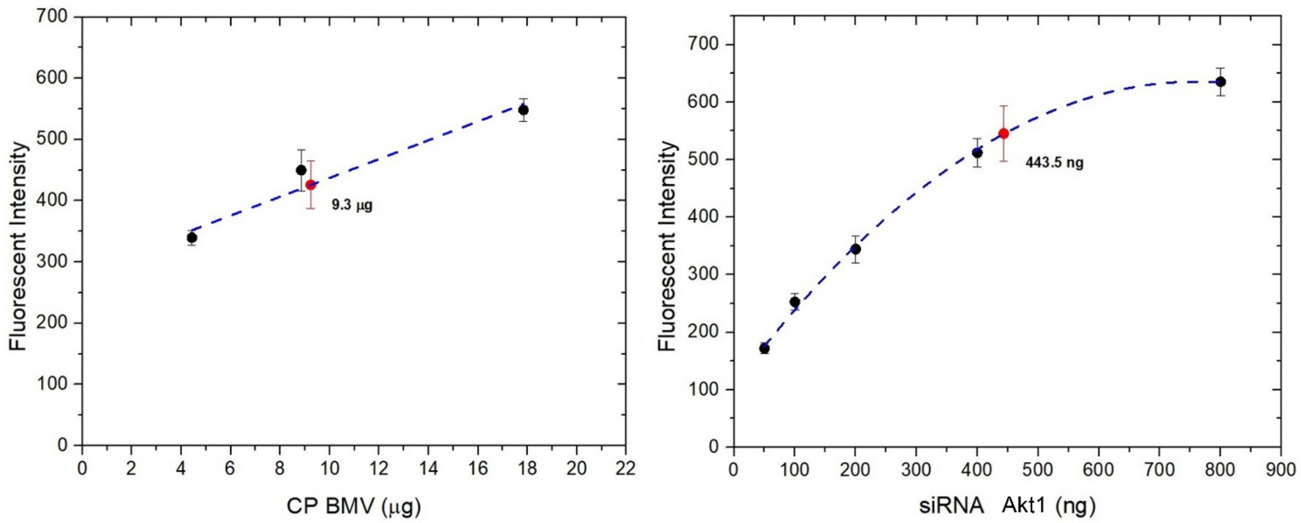

Figure S4: (a) BMV capsid protein fluorescent emission of three solutions of free capsid protein stained with NanoOrange and 1 µL of purified VLPs. (b) siAkt1 fluorescent emission of five solutions of free siRNA stained using Quant-iT RiboGreen RNA Assay Kit, and 5 µL of purified VLPs. According to the calibration curves, the BMV VLP-Akt1 solution has 100 µg of CP for every 23.8 µg of siRNA.
